# Prefrontal-Parietal-cTBS Effects on Metacognitive Awareness: Real or Illusory?

**DOI:** 10.1098/rsos.221193

**Published:** 2022-09-28

**Authors:** The Royal Society Open Science Editorial Team

**A. Stage 1 Title**: Prefrontal-Parietal-cTBS Effects on Metacognitive Awareness: Real or Illusory?

**B. Stage 1 Authors and Affiliations: Martin, Antonio**; Taipei Medical University, Graduate Institute of Mind, Brain and Consciousness (GIMBC); Shuang Ho Hospital, Brain and Consciousness Research Center (BCRC). **Lane, Timothy**; Taipei Medical University, Graduate Institute of Mind, Brain and Consciousness (GIMBC); Shuang Ho Hospital, Brain and Consciousness Research Center (BCRC) **Hsu, Tzu-Yu**; Taipei Medical University, Graduate Institute of Mind, Brain and Consciousness (GIMBC); Shuang Ho Hospital, Brain and Consciousness Research Center (BCRC).

**C. Stage 1 Abstract**: Neuroimaging and lesion studies suggest that the prefrontal parietal network (PPN) mediates visual metacognitive awareness. The causal evidence provided by noninvasive brain stimulation, however, is inconsistent. The activity of the PPN is linked to multiple processes, including metacognitive ratings of confidence and sensitivity, as well as reliability of expected sensory inputs. In order to shed light on which of the three alternatives is better supported by evidence, this study will use bilateral thetaburst transcranial magnetic stimulation over the PPN and a control site, for two experiments. One experiment uses a real figure discrimination task; the other, an illusory Kanizsa figure task. This study allows us to examine the three hypotheses and their relation to the PPN.

**D. Date of Stage 1 in-principle acceptance:** 9 February 2021

**E. Date of withdrawal:** 5 August 2022

**F. Reason for the withdrawal:** Due to COVID-19 and expiration of funding, the authors were unable to meet their sample size target, recruiting 20 of the required 62 participants specified in the approved protocol. An extension was offered by the journal but declined, and the authors are instead considering submitting their current results to another journal as a regular article.

**G. URL to the approved Stage 1 manuscript:** https://doi.org/10.17605/OSF.IO/RF29A.

